# Quality in crisis: a systematic review of the quality of health systems in humanitarian settings

**DOI:** 10.1186/s13031-021-00342-z

**Published:** 2021-02-02

**Authors:** Keely Jordan, Todd P. Lewis, Bayard Roberts

**Affiliations:** 1 Department of Health Policy, New York University School of Global Public Health, 665 Broadway, New York, NY 10012 USA; 2 Department of Global Health and Population, Harvard T.H. Chan School of Public Health, Boston, MA USA; 3 Department of Health Services Research and Policy, London School of Hygiene and Tropical Medicine, London, UK

**Keywords:** Global Health, Health policy, Humanitarian health, Crisis settings, Health systems

## Abstract

**Background:**

There is a growing concern that the quality of health systems in humanitarian crises and the care they provide has received little attention. To help better understand current practice and research on health system quality, this paper aimed to examine the evidence on the quality of health systems in humanitarian settings.

**Methods:**

This systematic review was based on the Preferred Reporting Items for Systematic Reviews and Meta-Analyses (PRISMA) protocol. The context of interest was populations affected by humanitarian crisis in low- and middle- income countries (LMICs). We included studies where the intervention of interest, health services for populations affected by crisis, was provided by the formal health system. Our outcome of interest was the quality of the health system. We included primary research studies, from a combination of information sources, published in English between January 2000 and January 2019 using quantitative and qualitative methods. We used the High Quality Health Systems Framework to analyze the included studies by quality domain and sub-domain.

**Results:**

We identified 2285 articles through our search, of which 163 were eligible for full-text review, and 55 articles were eligible for inclusion in our systematic review. Poor diagnosis, inadequate patient referrals, and inappropriate treatment of illness were commonly cited barriers to quality care. There was a strong focus placed on the foundations of a health system with emphasis on the workforce and tools, but a limited focus on the health impacts of health systems. The review also suggests some barriers to high quality health systems that are specific to humanitarian settings such as language barriers for refugees in their host country, discontinued care for migrant populations with chronic conditions, and fears around provider safety.

**Conclusion:**

The review highlights a large gap in the measurement of quality both at the point of care and at the health system level. There is a need for further work particularly on health system measurement strategies, accountability mechanisms, and patient-centered approaches in humanitarian settings.

**Supplementary Information:**

The online version contains supplementary material available at 10.1186/s13031-021-00342-z.

## Background

In 2015, the Sustainable Development Goals (SDGs) launched with the strong message of leaving no one behind [1]. These goals cannot be achieved without including the more than 70 million refugees, internally displaced persons (IDPs), and asylum-seekers [2]. The right to the highest attainable health extends to all individuals, regardless of their circumstance or legal status. International human rights treaties, most notably the International Covenant on Economic, Social and Cultural Rights, guarantee “the right of everyone to the enjoyment of the highest attainable standard of physical and mental health” [3].

There has been considerable progress in global health over the past 20 years driven by expanded access to public health services in low- and middle-income countries (LMICs) (e.g., clean water and sanitation) and health services (e.g., vaccination, antenatal care, and HIV treatment) [4]. These improvements have saved millions of lives primarily by averting deaths from infectious diseases [5]. However, strengthened strategies will be needed to tackle chronic and complex conditions and reach the health related SDGs. It has become clear that access alone is not enough without an accompanying focus on the quality of services being provided. High quality health systems are needed to improve health outcomes.

Humanitarian health activities commonly focus on immediate life-saving interventions. This often necessitates vertical programming in order to rapidly start and scale-up health services, particularly for historically key issues of preventing disease outbreak and reducing malnutrition. However, the challenge now is that crises are increasingly protracted, which requires longer-term and more coordinated and sustainable approaches. There are specific fears around the lack of continuity of care for conditions that require multiple visits to the health system, such as antenatal care (ANC) and postnatal care (PNC) and chronic conditions such as HIV/AIDS and diabetes [6]. This requires greater engagement in the broader health system and a focus on sustainable quality of care.

This review examined empirical evidence and provided an overview of how the literature defines and measures quality in crisis situations. Additionally, as a sub-aim, this review analyzed the methods used to measure quality (i.e., the quality of the quality measure), so that researchers can improve their work in this area. This review aimed to give direction to the humanitarian field on where future improvement strategies could be targeted. With the current emphasis in the global health community on quality of health systems this study is extremely relevant and timely.

### Defining high quality health systems

Building on the past work on quality and the developments in the field of health systems, the Lancet Global Health Commission on High Quality Health Systems in the SDG Era (HQSS) defined high quality health systems as systems that “optimize health in a given context by consistently delivering care that improves or maintains health, being valued and trusted by all people, and responding to changing population needs” [7]. This included, but was not limited to, the quality of care provided at the point of contact between provider and user. The definition goes on to state that equity, efficiency, and resilience are values that underpin a high quality health system [7]. Here, equity implies that it is available and affordable to all people regardless of underlying social disadvantages. An efficient system aims to achieve the highest possible health improvement with the given resources and a resilient system responds to routine and catastrophic challenges.

**Fig. 1:**
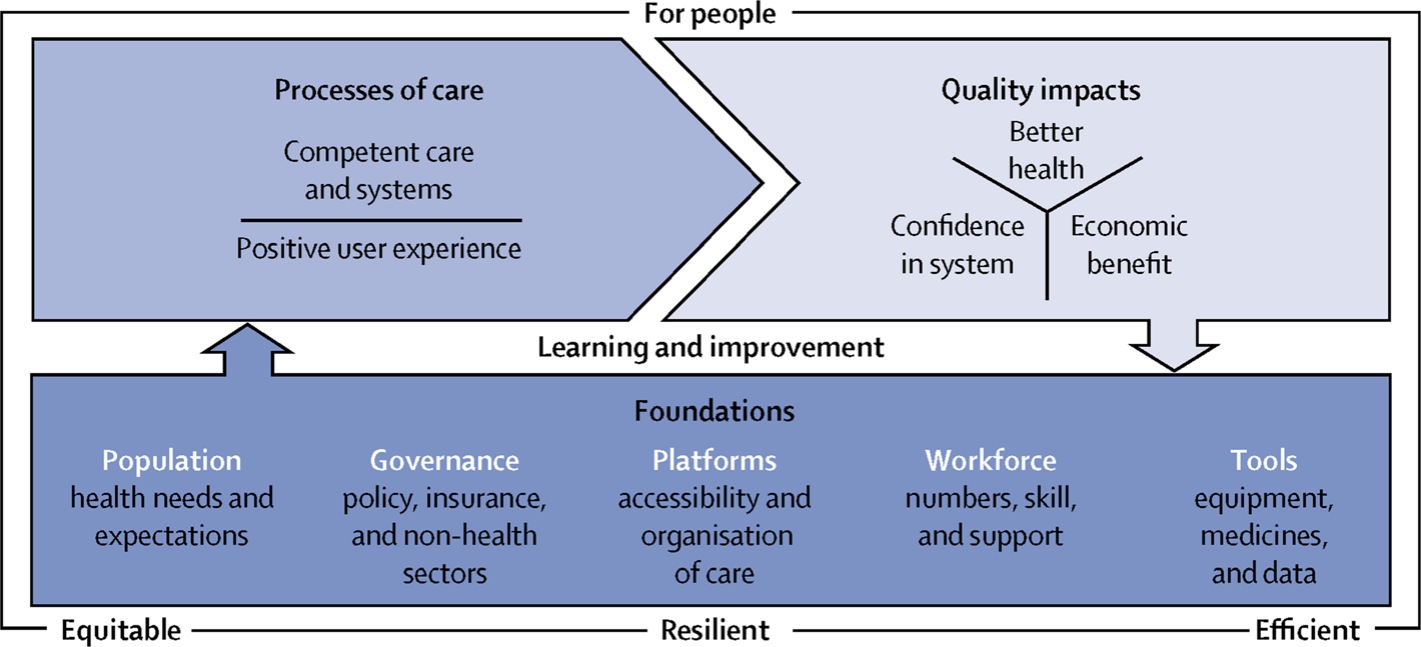
High Quality Health System Framework [7]

The conceptual framework developed by the HQSS Commission (Fig. 1) has three main domains: processes of care (competent care and user experience), quality impacts (better health, confidence in system and economic benefit) and foundations (population, governance, platforms, workforce and tools) [7]. The HQSS Commission believed that health systems should primarily be assessed based on processes and impacts of care because research shows that poor health systems can operate even when all the adequate tools are present [8]. The foundations of the system included the population’s health needs and expectations, governance of the health and non-health sectors, accessible and organized platforms of care, skilled workforce in numbers, and tools such as medicine and data.

### Health systems in crisis settings

Humanitarian crisis settings can vary widely in context, but are situations that involve widespread human suffering resulting from complex political, economic or social emergencies and natural hazards requiring large-scale provisions of aid. These include acute humanitarian crises that have a sudden onset and chronic, or protracted, humanitarian crises including forced displacement [9]. According to the *Lancet* Series on health in humanitarian crises, “protracted situations, often with additional acute emergencies, are becoming the new norm” [10]. Most refugees are not living in camps but rather in urban and rural areas and are not displaced but rather entrapped in conflict settings, such as those in Syria and Yemen. As a consequence, refugees and IDPs are at their highest number in over 50 years [11].

In many crisis situations, the health system goes through a period of degradation and fragmentation due to increasing violence and insecurity, weakening governance, and loss of resources. Reduced government activities create a void in services provided that is often filled by faith-based, private or informal providers [12]. Dozens of non-governmental organizations (NGOs) may be active in any of the main ongoing humanitarian crises and providing essential health services. These services tend to focus on primary health care for communicable disease control and management, nutrition, reproductive, maternal newborn and child health (RMNCH) and more recently mental health and NCDs. They are typically part of the UN Cluster System, with health care falling under the Health Cluster led by WHO, and sometimes in partnership with the local government [13]. They commonly follow the Sphere Standards and aim to support secondary and tertiary care facilities [14]. However, even within the same crisis different actors may pursue inconsistent and uncoordinated health strategies [15]. Assessing the performance of health actors in crises is challenging because of: insecurity and limited access; population mobility; short operational and funding time-periods (typically six-monthly cycles); rapidly developing health events; weak collection and sharing of routine health data; and limited monitoring, evaluation and research capacity [16].

This need has been magnified by the increasing number of conflicts in countries with a disease burden already heavily dominated by NCDs (e.g., Syria) [17]. To address these shortcomings there have been increasing efforts in the humanitarian sector to engage in accountability – particularly ensuring perspectives of affected populations are included [16]. In addition, despite the many epidemiological studies that have been conducted on humanitarian crises, there has been persistent concern over the quality of data for humanitarian crises [9, 18–23], and how data have been used to guide humanitarian health interventions and ultimately improve health outcomes [24–28]. To help better understand current practice and research on health system quality, this paper aims to examine the evidence on the quality of health systems in humanitarian settings.

## Methods

We conducted a systematic review based on the Preferred Reporting Items for Systematic Reviews and Meta-Analyses (PRISMA) protocol [29].

### Eligibility criteria

The context of interest was populations affected by humanitarian crisis in low- and middle- income countries (LMICs). We defined the situation of humanitarian settings using the criteria specified by the Sphere Standards [14] and the income status by the World Bank’s 2019 classifications. We included contexts affected by war, terrorist attack, political violence or armed conflict. Refugee/Internally displaced person (IDP) camps or settlements were also included, as were refugees, IDPs, or conflict-affected people who are living in non-camp settings. This included protracted displacement crises. We excluded large epidemics or pandemics since they vary considerably by pathogen and context. We also excluded post-conflict/post-disaster settings, which we classified as more than 5 years after the formal end of an armed conflict (e.g., signing of a peace agreement) or natural disaster.

We included studies where the intervention of interest, health services for populations affected by crisis, was provided by the formal health system. We defined the formal health system to be care by a trained provider, including public, NGO and private facilities. We excluded studies that fell outside the formal health system, for example school-based malnutrition campaigns.

Our outcome of interest was descriptive- how these studies defined and measured quality in the formal health system. We used the definition of high-quality health systems presented by HQSS and detailed above. We excluded studies if they reported solely on coverage or access to health care but did not specifically focus on quality.

We included primary research studies published in English between January 2000 and January 2019 using quantitative methods (any experimental, quasi-experimental, or observational design). We included randomized controlled trials if the outcome of interest was a quality measure but excluded clinical trials that aimed to prove only efficacy. Qualitative methods (any design) were included.

### Data sources and search terms

We used a combination of information sources to identify studies meeting the inclusion criteria: (1) electronic bibliographic databases for published studies, using a comprehensive search; (2) grey literature; and (3) the reference lists of studies included in the review. For the bibliographic databases, we searched published literature in PubMed, Embase, and Web of Science. For the grey literature, we searched the following databases and websites: WHO Global Health Library, UNHCR database, Reproductive Health Response in Crisis Consortium (RHRC), and the Inter-Agency Working Group on Reproductive Health in Crisis (IAWG). To approach literature saturation, we checked reference lists of included studies. We documented the information sources, including the name of each search, the date range searched and the search platform.

For the bibliographic database searching, we used Medical Subject Headings (MeSH) terms and key words from prior published literature. We constructed sets of search terms to capture four concepts: quality health system (which includes but is not limited to “quality of care”), focus on low- and middle-income countries, populations, and humanitarian settings (e.g., “quality” AND “LMIC” AND “population” AND “humanitarian setting”). The search strategy for PubMed is outlined in the Additional file 1: Appendix A. This strategy was applied to the other electronic databases and modified if necessary.

### Screening process

We downloaded and saved all search results into reference management software (EndNote version X7) and screened using an abstract management software (Abstrackr). Prior to the screening process we created a detailed selection criteria worksheet, which can be found in the Additional file 2: Appendix B, which all reviewers (KJ and TL) built consensus around and used through the screening process. We then did a pilot round applying the selection criteria to a subset of abstracts and came together to discuss the process. Two reviewers screened the entire set of abstracts (KJ and TL). We separately applied the criteria to the entire data set and adjudicated conflicts through consensus building. Full text reviews were done for the final sample by one reviewer (KJ) and reviewed by a second (TL).

### Data extraction and analysis

We applied the High Quality Health System Framework (Fig. 1) to help organize and analyze the data. This framework was chosen because it addresses health system quality more broadly, rather than just quality of care, is uniquely applicable to LMICs, and is easily adapted to humanitarian settings. Specifically, we extracted data related to the framework’s domains and sub-domains of quality health systems (Box 1).

**Box 1:** High Quality Health System Framework Domains and Sub-Domains [7]

Domain Sub-Domain	Definition
**Domain: Process of Care**	
Competent care and systems	Evidence-based, effective care: systematic assessment, correct diagnosis, appropriate treatment, counseling, and referral; capable systems: safety, prevention and detection, continuity and integration, timely action, and population health management
Positive user experience	Respect: dignity, privacy, non-discrimination, autonomy, confidentiality, and clear communication; user focus: choice of provider, short wait times, patient voice and values, affordability, and ease of use
**Domain: Quality Impacts**	
Better health	Level and distribution of patient-reported outcomes: function, symptoms, pain, wellbeing, quality of life, and avoiding serious health-related suffering
Confidence in system	Satisfaction, recommendation, trust, and care uptake and retention
Economic benefit	Ability to work or attend school, economic growth, reduction in health system waste, and financial risk protection
**Domain: Foundations**	
Population	Individuals, families, and communities as citizens, producers of better health outcomes, and system users: health needs, knowledge, health literacy, preferences, and cultural norms
Governance	Leadership; policies: regulations, standards, norms, and policies for the public and private sector, institutions for accountability, supportive behavioral architecture, and public health functions; financing; learning and improvement: institutions for evaluation, measurement, and improvement, learning communities, and trustworthy data
Platforms	Assets: number and distribution of facilities, public and private mix, service mix, and geographic access to facilities; care organization; connective systems: emergency medical services, referral systems, and facility community outreach
Workforce	Health workers, laboratory workers, planners, managers: number and distribution, skills and skill mix, training in ethics and people-centered care, supportive environment, education, team work, and retention
Tools	Hardware: equipment, supplies, medicines, and information systems; software: culture of quality, use of data, supervision, and feedback

We extracted data from the final eligible studies using a standardized form. The following variables were extracted: year of publication, title, study type, evaluated country, study setting, population type, number of participants, study description, quality domain (from framework, Box 1), quality sub-domain (from framework, Box 1), methodological quality, main findings/results, miscellaneous. A deductive approach was used to organize the analysis within the domains of the High Quality Health System Framework and then an inductive approach used to explore emerging themes from within the domains of the framework.

To assess the methodological quality of the included studies, we evaluated each study in six domains: selection bias, appropriateness of data collection, appropriateness of data analysis, generalizability, ethical considerations and clarity of the study’s methods [30]. This domain-based evaluation was chosen instead of a scale or checklist due to its ability to critically assess different domains separately, as suggested by The Cochrane Collaboration [31].

## Results

**Fig. 2:**
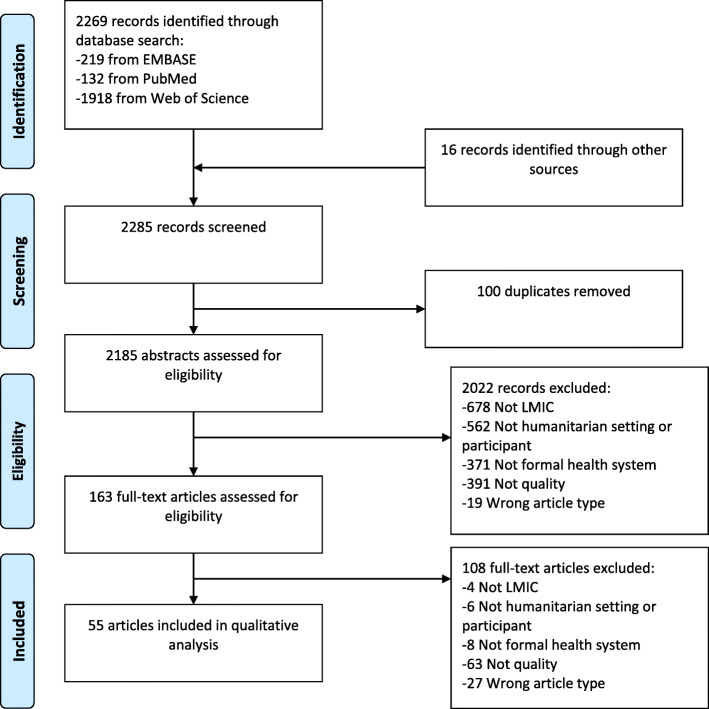
Flowchart showing the selection of studies

We identified 2269 records through the database search and an additional 16 records through the other sources. After removal of duplicates and screening, 55 papers remained for full-review (the screening process and main reasons for exclusion are provided in Fig. 2).

Of the 55 papers included in the systematic review the majority of studies were published since 2015 (34; 62%). Multiple study designs were used: 17 (31%) were quantitative, 16 (30%) were qualitative, and another 13 (24%) used mixed quantitative and qualitative methods. Eleven (20%) took place in the WHO African Region, two (4%) in the European Region, eight (14%) in the South East Asia Region, 25 (45%) in the Eastern Mediterranean Region, two (4%) in the Western Pacific Region, and seven (13%) were multi-country studies that spanned multiple regions. The majority of studies took place in conflict-affected settings (45; 82%), eight (14%) in refugee settlements, and only two (4%) studies taking place in a post-natural disaster setting. About one third of the population type studied was refugees, IDPs, or conflict-affected cross-boarder migrants (17; 31%). Fifteen studies (27%) focused on women and/or children, and 11 (20%) focused solely on health care providers. Table 1 describes the characteristics of included studies.

**Table 1:** Characteristics of included studies

Study Characteristics	No. (%) of studies (***n*** = 55)
**Year**	
2000–2009 [32–37]	6 (11)
2010–2014 [38–52]	15 (27)
2015–2019 [53–86]	34 (62)
**Study Type**	
Quasi-experimental [38, 41, 68]	3 (5)
Cohort [49, 65]	2 (4)
Cross-sectional [36, 39, 43, 53, 62–64, 77, 81–83]	11 (20)
Mixed methods [37, 45, 46, 48, 57, 60, 69, 71, 72, 75, 80, 85, 86]	13 (23)
Qualitative [33, 42, 44, 47, 50, 56, 59, 61, 66, 67, 73, 74, 76, 78, 79, 84]	16 (30)
Case study [32, 34, 35, 40, 51, 52, 54, 55, 58, 70]	10 (18)
**WHO Region**	
Americas	0
African [33, 36, 41, 53, 59, 62, 67, 68, 81, 85, 86]	11 (20)
European [64, 82]	2 (4)
South-East Asia [35, 38, 44, 45, 49, 51, 60, 73]	8 (14)
Eastern Mediterranean [32, 37, 39, 40, 42, 46–48, 52, 55, 56, 61, 63, 65, 66, 69–71, 74, 75, 77–80, 83]	25 (45)
Western Pacific [34, 84]	2 (4)
Multiple Regions [43, 50, 54, 57, 58, 72, 76]	7 (13)
**Setting**	
Conflict-affected (including IDPs and cross-boarder migrants) [32–34, 36, 37, 39–42, 44, 47–59, 61, 63–71, 73–75, 77–80, 82–86]	45 (82)
Refugee settlements [43, 45, 46, 60, 62, 72, 76, 81]	8 (14)
Natural disaster [35, 38]	2 (4)
**Population Type**	
Women and/or children [34, 43, 45, 48, 49, 57, 60, 66, 68, 70, 71, 75, 77–79]	15 (27)
Refugees/IDPs/conflict-affected cross-boarder migrants [32, 33, 35, 36, 38, 50, 62, 65, 67, 69, 72, 73, 80–84]	17 (31)
Health care providers [40, 42, 44, 46, 51, 56, 58, 61, 64, 74, 76]	11 (20)
General population [37, 39, 41, 52, 53, 55, 85, 86]	8 (14)
Patients and providers [47, 54, 59, 63]	4 (8)

We intentionally did not restrict the papers included by study design in order to gain insight from qualitative and mixed methods studies. The rigor of the studies was assessed and the majority (34, 62%) of the studies adequately addressed at least five of the six quality areas. Fifty-one (93%) studies used a sample that was appropriate to its research questions, 49 (89%) studies collected the data appropriately, 46 (84%) analyzed the data appropriately, 31 (56%) studies had results that were transferable by setting, 23 (42%) adequately addressed potential ethical issues, and 48 (87%) studies were clear in their overall approach.

We used the High Quality Health Systems Framework to analyze the included studies by quality domain and sub-domain. The majority of studies (46; 84%) addressed multiple quality domains simultaneously, with a strong emphasis on workforce (38; 69%), tools (26; 45%), and competent care (23; 44%). Table 2 depicts the numbers of addressing the domains and sub-domains.

**Table 2:** Quality domains addressed in included studies

Domain Sub-Domain	Studies
**Domain: Process of Care**	
Competent care [32, 34, 37, 40, 41, 43, 44, 46, 49, 51, 57, 59, 60, 65, 66, 68–70, 75–77, 81, 83]	23
Competent systems [35, 41, 43, 45, 66, 69, 72, 73, 76, 82, 85]	11
Positive user experience [33, 36, 39, 41, 45, 47, 50, 59, 62, 64, 67, 72, 75, 79]	14
**Domain: Quality Impacts**	
Better health [34, 49, 54, 65, 77, 81, 84]	7
Confidence in system [32, 33, 35, 39, 53, 62, 67, 78, 83]	9
Economic benefit [84–86]	3
**Domain: Foundations**	
Population [45, 53, 58, 67, 73, 79, 84–86]	9
Governance [34, 38, 52, 55, 61, 80, 84–86]	9
Platforms [39, 52, 57, 70, 81, 85]	6
Workforce [33, 34, 37, 38, 40, 42, 43, 45–49, 51, 52, 56–61, 63, 64, 66, 67, 69–72, 74–76, 78–81, 84–86]	38
Tools [32, 34, 38, 39, 41, 42, 45, 48, 52, 54, 57–61, 63, 64, 70, 72, 79, 81, 83–86]	26

### Process of care

The majority of the studies included addressed process of care as a quality domain. A theme that emerged within those studies was that poor diagnosis and treatment was a major barrier health systems faced in conflict settings. Common manifestations of poor quality were incorrect diagnosis [37, 40, 44, 66, 69] and inappropriate treatment of illness [44, 46, 73, 81, 82]. Lainez and colleagues highlighted the issue of “competent care” in their study on the prevalence of respiratory symptoms in Afghanistan and found that there was a gap in diagnosis with 23.8% of patients with TB-suggestive symptoms going undiagnosed [37]. Another manifestation of the “competent care” sub-domain was necessary patient referrals [32, 34, 66, 69, 77]. For example, Elmusharaf et al. found that outcomes were better for pregnant women in South Sudan where there was no facility available rather than when the woman accessed a non-functioning facility, and the absence of a health care provider was better than the presence of a non-competent provider [66]. This finding was primarily influenced by inadequate referral systems (including late referrals to appropriate facilities and multiple referrals). In two studies the lack of continuity of care was expressed as a quality concern [35, 66]. However, when care was integrated (e.g., mental health services into primary health care) a positive outcome was seen [40, 76].

User experience, including feeling that the health staff was judgmental or discriminatory was cited as a major barrier to high quality care and impacted care seeking behavior [33, 36, 41, 47, 67]. For example, the study by Kruk and colleagues on population preferences for health care in Liberia showed that a patient’s choice of clinic was influenced by respectful treatment along with other factors such as provision of a thorough physical exam, availability of medicines, and government management [41]. Language barriers and the lack of clear communication also negatively impacted the patient-provider relationship in the studies examined [33, 45, 50, 67, 72, 75]. This was particularly true for refugees in host countries [33, 45, 50, 72].

### Quality impacts

About one third of included studies addressed “quality impacts” as a quality domain. Nine focused on the patient’s confidence in the system, seven focused on better health, and three focused on economic benefit.

The patient’s confidence in the health system was most commonly measured as care satisfaction [35, 39, 47, 53, 62, 78, 79, 83]. The findings around satisfaction were mixed. Some studies reported that patients were dissatisfied with the low quality care they received [47, 78, 79], and Kibiribiri et al. found that refugees were more dissatisfied than the general population in South Africa [62]. However, other studies reported that patient satisfaction was high even where quality was poor [35, 39, 83]. In their study on perceptions and utilization of primary health care services in Iraq, Burnham and colleagues showed that high satisfaction corresponded with low expectations of the health system [39]. The patient’s perception of the low quality of care was a barrier to care uptake and retention in the health system [32, 67, 78]. A qualitative study by Hunter-Adams et al. on the language barriers between South African health care providers and conflict-affected cross-boarder migrants suggested that providing interpretive services could increase the patient’s confidence in the system and potentially increase preventative care visits [67].

In many studies, clinical outcomes, most commonly hospital mortality, were used to measure the quality of care provided by the health system [34, 43, 49, 54, 65, 75, 77]. For example, Auto et al. discussed the importance of hospital quality improvement strategies to start with a clear understanding of (child) mortality, its causes, and distribution.

The economic burden that poor quality care had both on the patient and the system was only addressed in three studies [84–86]. Chuah and colleagues used a qualitative approach to assess the health system responses to the health needs of refugees and asylum-seekers in Malaysia. One of the key findings was that healthcare financing was a major challenge in responding to refugee health issues and even with the discounted fee for refugee patients at public healthcare facilities the out-of-pocket expenditure for them was still too high [84]. Bertone et al. examined how performance based financing (PBF) can be adapted in fragile settings and found that providing free care to IDPs, even where free care was not an official government policy, successfully improved access by reducing financial burden [86]. Additionally, a study on health service resilience in Nigeria found that political instability had a direct impact on financial barriers, which through multiple pathways influenced utilization of health services [85]. This study found that drug subsidy schemes and programs offering free services “moderated the health impact of the disruption of livelihoods resulting from insurgency” [85].

### Foundations

The majority of the studies included addressed the foundations of a health system. A common theme that emerged was an emphasis on the workforce and tools quality sub-domains. The evidence identified on workforce constraints focused on human resource shortages [34, 39, 57, 63, 80, 81, 84], low workforce moral [47, 75], and inadequate provider training [48, 51, 56, 58, 61, 71, 74] as the most cited causes of poor quality. A qualitative study in Afghanistan articulated the intense physical and mental pressures that medical staff face in the field and assured that sub-optimal care was unlikely deliberate but rather the result of “conflicting priorities, the workload, poor clinical skills and the struggle for survival” [56]. The physical resources needed for a functioning health system was often used in the studies as a quality measure, specifically limited access to medicines [32, 34, 38, 57, 60, 61, 79, 81, 84] and supplies [42, 48, 57–60, 63, 64, 70, 72, 79]. Mowafi and colleagues highlighted the severe material and human resource constraints that Syrian trauma hospitals operate under including the large amount of nonfunctioning diagnostic equipment (e.g., 23% broken X-ray machines) [63].

Other quality sub-domains were also addressed, though to a lesser degree. Within the “population” sub-domain, the cultural barriers patients faced during service delivery were raised in a number of studies [45, 53, 58, 67, 73, 79, 84, 85]. In a study on refugee and migrant women’s views of antenatal ultrasound, Rijken and colleagues suggested that “transient embarrassment or shame on exposing the abdomen (a part not normally exposed in public by local women in this culture)” was a potential barrier to receiving medical attention [45]. Community resources and cohesion, as described by Ager et al., was identified as a key driver of pathways of influence mitigating the impacts of the crisis. The study went on to detail how communities “pooled resources (knowledge, transport and finance) to enable physical and financial access to health facilities for those in need”. Communities also played a key role in mobilizing political will for quality care [85]. “Platform” barriers to quality care were included in six studies and addressed the number and distribution of facilities [39, 52, 57, 85] and the disorganized structure of service delivery [70, 81]. A study looking at the implementation of Afghanistan’s Basic Package of Health Services (BPHS) found that access to and utilization of primary health care services in rural areas increased dramatically because the number of BPHS facilities more than doubled [52]. Governance improvements, specifically political commitment and enhanced leadership, were highlighted as necessary ways to improve health system performance [34, 38, 52, 55, 61, 80, 84–86]. A study in Afghanistan by Anwari et al. suggested that improvements in stakeholder engagement, cultivating accountability, setting a shared strategic direction and stewarding resources responsibility were possible by implementing a people-centered governance approach [55].

## Discussion

This systematic review, which included 55 studies, examined the evidence on the quality of health systems providing care in humanitarian settings. It was the first study to our knowledge that addressed this topic. The key findings suggest: poor diagnosis and inappropriate treatment of illness (including inadequate patient referrals) were commonly cited barriers to quality care; there was a limited focus on the health impacts that health systems have in the studies identified; and a strong focus was placed in these studies on the foundations of a health system with emphasis on the workforce and tools. The implications from these findings for future research include: expanding the definition of quality to include quality impacts, developing and validating quality measures suitable for crisis settings, and incorporating more diverse and rigorous study designs.

There are many contextual challenges highlighted in the identified literature to providing quality care in humanitarian settings, however, there are also findings from this review that are broadly in line with evidence from more stable LMICs. In terms of “process of care” the HQSS Commission also found that many LMICs struggle to consistently deliver high quality care and well-known, effective treatments are not consistently provided [7]. Many of the studies included in this review attempted to improve care competence with short in-service provider trainings. However, there are many studies that attest to the know-do gap (i.e., the gap between provider knowledge and clinical care provided) [87, 88], suggesting that these efforts may not have the long-term quality improvements they are aiming for. Further, the disrespectful care that many of the studies attested to is widespread throughout LMIC health systems. The HQSS Commission found that 1 in 3 patients experienced disrespectful care, short consultations, poor communication or long wait times [7].

The limited focus that the studies included in this review placed on “quality impacts” belies the primary goal of health systems, which is to improve or maintain health. The initial findings from this review suggest potentially lower levels of satisfaction in crisis settings than the general population. High satisfaction with health care is common across LMICs even where quality is poor, possibly due to low expectations, and the HQSS Commission warned that patient satisfaction as a measure of quality should be carefully interpreted. A lack of confidence in the health system can in turn hinder care uptake and retention, which is already fragile given the setting. Although causes of death and disease are multifactorial some conditions are highly dependent on quality of care and how well the health system is working, such as maternal and newborn deaths [89]. The emphasis that the studies in this review placed on measuring maternal, newborn, and child mortality is therefore in line with other LMIC health system quality indicators. High quality health systems generate many economic benefits, such as reducing premature mortality and reducing health system waste. The studies in this review, however, primarily forced on financial risk protection [85, 86].

The “foundations” of a health system are often the most cited and measured elements of quality [7, 90]. Workforce and facility constraints, specifically, are widespread through LMICs and the HQSS Commission found that 47% of improvement research was targeted towards these two sub-domains [7]. Though foundations are essential to health-care provision, prior studies have commented on the weak associations between input measures and care competence [8]. The HQSS Commission therefore recommended a shift in measurement away from foundations (or inputs) to what matters most to people: competent care, user experience, health outcomes, and confidence in the system [7]. This people-centered approach places the emphasis on the user of the health system and aims to create a setting in which people have agency over their own health and health-care decisions. This can be particularly challenging in the face of violence, displacement or forced migration. Strengthening health system quality has unique challenges in crisis situations, particularly when the majority of services are provided by NGOs and large gaps exist in critical services, and inequity in the distribution of those services. The studies in this review suggest that a key step is assessing the needs of the population in crisis, who are often vulnerable groups that face further marginalization due to context. Keeping equity and the underserved a priority from the beginning requires localizing health system improvements. The low emphasis that the studies placed on “population” needs suggest it is an area to focus for improvement. As many of the studies in this review point out changes towards a people-centered health system take strong local political commitment and leadership. In support of this finding, the WHO National Quality Policy and Strategy Handbook suggested that well-aligned policies and strategies be based on locally-accepted definitions of quality and national goals for improved outcomes [91].

The findings from this review are in line with the broader literature on humanitarian settings and highlight the limited evidence on health impacts of interventions and use of economic methods in humanitarian settings [92, 93]. There were many studies that fell outside the eligibility criteria of this review that assessed the role of user fees and performance incentives on quality and utilization rates that could offer lessons and areas for future study [94–96]. Additionally, there is a call to increase measurement in humanitarian settings and link that to a focus on accountability mechanisms [6, 16]. Different quality measurement techniques have been used in humanitarian contexts but an overarching theme was that consideration and adaption of design processes are needed to meet local circumstances [6, 97]. Process of care measures (i.e., what a provider does to maintain or improve health) have shown to play an important role in assessing care for vulnerable populations [98] and this review suggests they could be particularly useful to inform the users about the care they should expect to receive and increase demand for high quality. There are additional measurement constraints in crisis settings due to insecurity and other contextual challenges and resource constraints, which in many instances make more rigorous study designs, particularly experimental studies, operationally and ethically challenging [9, 99, 100]. There are, however, many valuable studies on health system resilience and rebuilding in post-conflict and fragile settings that could be useful in humanitarian contexts [101, 102]. Also, alternate designs have proven successful in humanitarian settings at showing changes in health outcomes over time, such as stepped wedge designs, greater use of longitudinal data and routine health services data, and collection of process data that can be a reliable proxy for health outcome data [9, 103, 104]. Additionally, due to the complexity of health systems research a more narrow approach that focuses on specific aspects of quality may be beneficial for future work as well as a stronger use of interdisciplinary research (e.g., social science, political science, epidemiology).

The findings from this study have many policy relevant implications and they point to the need for ‘macro-level’ system wide transformations. First, they highlight the need for new and improved quality measurements that move beyond the foundational aspects of quality. Adopting measures that focus on system competence (e.g., timeliness and continuity of care), user experience and health outcomes could potentially shape future quality improvement strategies. Second, they point to a need for initiatives that focus on improving accountability in humanitarian settings. Multipronged strategies that build partnerships across the system are needed that combine legal, performance and social accountability tools. This potentially involves legislating for vulnerable people’s right to quality health care, educating the population on their rights, creating strong regulations and standards, and enforcing mechanisms for remedy and redress. Finally, they suggest that there has been a failure to respond to key health system concepts such as patient-centered care. All people deserve to be treated with respect and dignity within the health system. Additionally, health workers need to receive the support they need to fulfill their professional duty even under the most dire circumstances. A high-quality people-centered health system should take into account the needs, experiences, and preferences of even the most vulnerable populations.

## Limitations

We may not have captured all the data available on quality of care in humanitarian settings. In particular, we limited our review to English language studies, only used three electronic bibliographic databases, and though the entire first round was double screened only one screener did the final full text reviews. The absence of experimental designs is also a limitation considering they could show attribution of interventions to changes in health outcomes. However, this review included a large number of qualitative and mixed-methods studies, which provides a unique insight into user experience.

The two areas of methodological quality in which included studies were lacking were generalizability and ethical considerations. In terms of generalizability, most of the studies included in our review were on conflict-affected settings and the findings may not be applicable to other crises such as natural disasters. There are many ethical challenges when doing research in humanitarian crises considering the added vulnerability of the population.

## Conclusion

There has been a growing interest on the quality of care health systems provide in humanitarian settings throughout the past two decades. However, a large gap still exists on studies that systematically measure quality both at the point of care and at the health system level. The findings from this review highlight key quality issues including incorrect diagnoses and treatments, low levels of confidence in the health system, and a disproportional emphasis on the health workforce and tools. The review also suggests some barriers to high quality health systems that are specific to humanitarian settings such as language barriers for refugees in their host country, discontinued care for migrant populations with chronic conditions, and fears around provider safety. Individuals, families, and communities in humanitarian crises have specific health needs that require an understanding of their culture, preferences, and health knowledge in order to be met. There is need to expand work on the topic, particularly for focusing on health system measurement strategies, accountability mechanisms, and patient-centered approaches in humanitarian settings.

## Supplementary Information

**Additional file 1.**

**Additional file 2.**

## Data Availability

The data that support the findings of this study are available from the corresponding author upon reasonable request.
