# Supplementary material for: Quality in crisis: a systematic review of the quality of health systems in humanitarian settings
Source: Confl Health. 2021 Feb 2;15:7. doi: 10.1186/s13031-021-00342-z (PMC7851932; doi:10.1186/s13031-021-00342-z)
Supplement: Supplementary file 1 — Additional file 1. [file 13031_2021_342_MOESM1_ESM.docx]

**Appendix A: Search Strategy**

Population Hedge:

Refugees OR refugee OR asylum seeker OR Internally Displaced Population OR Internally Displaced Person OR IDP OR displaced population OR displaced person OR displaced OR conflict affected OR war affected OR war exposed OR war population

Humanitarian Settings Hedge:

Armed conflicts OR ethnic violence OR war exposure OR terrorism OR genocide OR human migration OR relief work OR altruism OR disasters OR refugee camp OR armed conflict OR post-conflict situation OR post-conflict setting OR conflict OR post conflict OR war OR war conflict OR mass conflict OR mass killing OR terrorist attack OR political violence OR ethnocide OR genocide OR ethnic cleansing OR mass violence OR forced migration OR humanitarian crisis OR humanitarian setting OR complex humanitarian setting OR humanitarian emergency OR complex emergency OR Complex Humanitarian Emergencies OR CHEs OR CHE OR refugee camp OR refugee setting OR camp OR settlement OR mass casualty OR displacement

Quality Hedge:

Standard of Care OR Program Evaluation OR Patient Satisfaction OR patient-centered care OR Organizational Case Studies OR Evaluation Studies as Topic OR Guideline Adherence OR Clinical Competence OR Utilization Review OR Quality Indicators OR Quality Improvement OR Culturally Competent Care OR Delivery of Health Care, Integrated OR Professional-Patient Relations OR Quality Assurance OR Outcome Assessment OR Process Assessment OR evidence-based practice OR patient reported outcome measures OR patient harm OR patient safety OR Quality of Care OR Standard of Care OR Standards of Care OR Care Standard* OR Patient Satisfaction OR Patient Preference* OR patient-centered care OR Patient centered care OR patient-focused care OR patient focused care OR patient-centered nursing OR patient centered nursing OR Outcome and Process Assessment OR Structure Process Outcome OR Donabedian model OR Donabedian Triad OR Organizational Case Stud* OR Management case stud* OR Guideline Adherence OR Policy Compliance OR Protocol Compliance OR Clinical Competenc* OR clinical skill* OR Utilization Review* OR Global trigger tool OR Quality Improvement* OR Cross-cultural care OR Cross cultural care OR Cultural competen* OR Integrated Delivery of Health Care OR Integrated Delivery of Healthcare OR Integrated Health Care Systems OR Integrated Healthcare Systems OR Professional-Patient Relation* OR Professional Patient Relation* OR Nurse-patient relation* OR Nurse-patient relation* OR Doctor-patient relation* OR Doctor patient relation* OR Physician-patient relation* OR Physician patient relation* OR Clinician-patient relation* OR Clinician patient relation* OR patient reported outcome* OR patient harm* OR patient safet*OR Quality of Care OR Healthcare Quality OR health care quality OR care qualit* OR Quality of healthcare

LMIC Hedge:

Developing Countries OR developing countr* OR under developed countr* OR developing nation* OR developing world OR less developed world OR lmic* OR lower income OR middle income OR low middle income OR resource poor OR resource constrained OR low resource OR limited resource* OR Africa South of the Sahara OR Central America OR South America OR Latin America OR Caribbean Region OR Mexico OR Asia OR Afghanistan OR Afghan OR Albania* OR Algeria* OR American Samoa* OR Angola* OR Argentina OR Argentinian OR Armenia* OR Azerbaijan* OR Bangladesh* OR Belarus OR Belorussian OR Beliz* OR Benin* OR Bhutan* OR Bolivia* OR Bosnia OR Bosnian* OR Herzegovin* OR Botswan* OR Brazil OR Brazilian OR Bulgaria* OR Burkina Faso OR Burkinabe OR Burma OR Burmese OR Burund* OR Cambodia* OR Cameroon* OR Cape Verde OR Cape Verdean OR Central African Republic OR Chad OR Chadian OR China OR Chinese OR Colombia OR Colombian OR Comoros OR Comorian OR Congo OR Congolese OR Costa Rica OR Costa Rican OR Côte d’Ivoire OR Ivory Coast OR Ivorian OR Cuba OR Cuban OR Djibouti* OR Dominica OR Dominican OR Ecuador* OR Egypt OR Egyptian OR El Salvador OR Salvadorian OR Guinea OR Guinean OR Guinea-Bissau* OR Eritrea* OR Ethiopia* OR Fiji* OR Gabon* OR Gambia* OR Gaza OR Georgia OR Georgian OR Ghana OR Ghanaian OR Grenad* OR Guatemala* OR Guyan* OR Haiti* OR Hondura* OR India OR Indian OR Indonesia* OR Iran OR Iranian OR Iraq* OR Jamaica* OR Jordan OR Jordanian OR Kazakh* OR Kenya OR Kenyan OR Kiribati OR Korea* OR Kosov* OR Kyrgyz Republic OR Kyrgyzstan OR Laos OR Laotian OR Lebanon OR Lebanese OR Lesotho OR Liberia* OR Libya* OR Macedonia* OR Madagasca* OR Malawi* OR Malaysia* OR Maldives OR Maldivian OR Mali OR Malian OR Marshall Islands OR Mauritania* OR Mauritius OR Mauritian OR Mexico OR Mexican OR Micronesia* OR Moldov* OR Mongolia* OR Morocc* OR Mozambique OR Mozambican OR Myanmar OR Namibia* OR Nepal* OR Nicaragua* OR Niger OR Nigeria* OR Pakistan* OR Palau* OR Panama* OR Papua New Guinea OR Paraguay* OR Peru* OR Philippine* OR Romania* OR Russia* OR Rwanda* OR Samoa* OR Sao Tome OR Senegal* OR Serbia OR Serbia* OR Montenegr* OR Sierra Leone OR Slovak Republic OR Slovakia* OR Solomon Islands OR Somali* OR South Africa OR South African OR Sri Lanka OR Sri Lankan OR Saint Kitts OR Saint Lucia OR Saint Vincent OR Sudan* OR South Sudan* OR Suriname* OR Swaziland OR Swazi OR Syria OR Syrian OR Tajikistan OR Tajik OR Tanzania* OR Thailand OR Thai OR Timor‐Leste OR Togo* OR Tonga* OR Tunisia* OR Turk* OR Turkmenistan OR Tuvalu* OR Uganda* OR Ukrain* OR Uzbekistan OR Uzbek OR Vanuat* OR Venezuela* OR Vietnam* OR West Bank OR Yemen* OR Zambia* OR Zimbabwe*
