# Supplementary material for: Quality in crisis: a systematic review of the quality of health systems in humanitarian settings
Source: Confl Health. 2021 Feb 2;15:7. doi: 10.1186/s13031-021-00342-z (PMC7851932; doi:10.1186/s13031-021-00342-z)
Supplement: Supplementary file 2 — Additional file 2. [file 13031_2021_342_MOESM2_ESM.docx]

**Appendix B: Selection Criteria**

INCLUDE IF ARTICLE:

- Was published in English in PubMed, Web of Science, or Embase between 2000-2019
- Study takes place in LMIC
- Includes humanitarian setting and participants: contexts affected by war, terrorist attack, political violence, armed conflict or natural disasters. Refugee/IDP camps or settlements are eligible settings. All refugees, IDPs, or conflict-affected people who are living in non-camp settings (e.g urban or rural area) are included as well.
- Involves the formal health system: organized health sector, public and private, including community health workers but not informal providers
- Includes quality of care: care that improves or maintains health outcomes, by being valued and trusted by all people, and by responding to changing population needs (i.e. for people, equitable, resilient, efficient)
  - Component care and systems
  - Positive user experience
  - Better health
  - Confidence in system
  - Economic benefit
  - Foundations (population, governance, platforms, workforce, tools)

EXLUCDE IF ARTICLE:

- Takes place in high-income country (including studies on refugee populations in HIC) **(Tag 1)**
- Does not include humanitarian setting or participants (including military hospitals or veterans) **(Tag 2)**
- Care is provided outside formal health system by informal provider with little or no formal clinical training **(Tag 3)**
- Does not include quality of care, as evidenced by the following: **(Tag 4)**
  - Solely reports on coverage of health service
  - Solely reports on access to health care
- Is a case report, review article, editorial, commentary, perspective, personal narrative, not full-length, not published in English **(Tag 5)**

TAGS FOR REVIEWING:

1. Not LMIC
2. Not humanitarian setting or participant
3. Not formal health system
4. Not quality of care
5. Wrong article type
